# Scaled Synthesis of Polyamine-Modified Cellulose Nanocrystals from Bulk Cotton and Their Use for Capturing Volatile Organic Compounds

**DOI:** 10.3390/polym13183060

**Published:** 2021-09-10

**Authors:** Beau R. Brummel, Chandima J. Narangoda, Mohamed F. Attia, Maria I. Swasy, Gary D. Smith, Jr., Frank Alexis, Daniel C. Whitehead

**Affiliations:** 1 Department of Chemistry, Clemson University, Clemson, SC 29634, USA; bbrumme@g.clemson.edu (B.R.B.); cnarang@g.clemson.edu (C.J.N.); mattia@clemson.edu (M.F.A.); mswasy@g.clemson.edu (M.I.S.); 2 Baker Commodities, Inc., Vernon, CA 90058, USA; dsmith@bakercommodities.com; 3 School of Biological Sciences and Engineering, Yachay Tech University, Urcuqui 1000650, Ecuador

**Keywords:** cellulose nanocrystals, polyamines, poly(ethylenimine), odor, VOCs, volatile fatty acids

## Abstract

We have previously demonstrated that cellulose nanocrystals modified with poly(ethylenimine) (PEI-*f*-CNC) are capable of capturing volatile organic compounds (VOCs) associated with malodors. In this manuscript, we describe our efforts to develop a scalable synthesis of these materials from bulk cotton. This work culminated in a reliable protocol for the synthesis of unmodified cellulose nanocrystals (CNCs) from bulk cotton on a 0.5 kg scale. Additionally, we developed a protocol for the modification of the CNCs by means of sequential 2,2,6,6-tetramethylpiperidine 1-oxyl (TEMPO) oxidation and 1-ethyl-3-(3-dimethylaminopropyl)carbodiimide hydrochloride (EDC) coupling to modify their surface with poly(ethylenimine) on a 100 g scale. Subsequently, we evaluated the performance of the PEI-*f*-CNC materials that were prepared in a series of VOC capture experiments. First, we demonstrated their efficacy in capturing volatile fatty acids emitted at a rendering plant when formulated as packed-bed filter cartridges. Secondly, we evaluated the potential to use aqueous PEI-*f*-CNC suspensions as a spray-based delivery method for VOC remediation. In both cases, the PEI-*f*-CNC formulations reduced detectable malodor VOCs by greater than 90%. The facile scaled synthesis of these materials and their excellent performance at VOC remediation suggest that they may emerge as a useful strategy for the remediation of VOCs associated with odor.

## 1. Introduction

The development of functional nanomaterials for environmental applications is a burgeoning field of study [1]. Leveraging renewable materials as a platform for the development of environmental remediation strategies is a particularly appealing endeavor. Nanocellulose-based materials [2] have enjoyed wide applicability in this space, and represent a promising renewable feedstock for the development of sustainable platforms to address environmental needs [3,4,5,6]. More specifically, polyamine-decorated cellulose materials and related formulations have been explored for a number of environmental applications related to wastewater including the removal of anionic dyes [7,8,9,10,11], metal ions [11,12,13,14,15], and other environmental contaminants [16,17]. Despite the utility of nanocellulose for such applications, there are currently rather few raw material sources—wood pulp and marine tunicates—that provide access to CNCs at the kilogram scale [18]. 

Our laboratory has had a long-standing interest in exploring functional nano- and micro-scale materials for environmental remediation [19,20,21,22,23,24,25,26,27]. Recently, we developed a route to access poly(ethylenimine)-modified cellulose nanocrystals (PEI-*f*-CNC) that are capable of capturing volatile organic compounds associated with odors [22] and degrading common pesticides from water [27]. Related poly(amine)-modified cellulose microcrystals (i.e., PEI-*f*-CMCs) were effective for the removal of polyfluorinated surfactants from water [21].

In this manuscript, we describe a convenient and readily scalable synthesis of cellulose nanocrystals (CNCs) from bulk cotton and a scaled synthesis of PEI-*f*-CNC from in-house prepared CNCs. Furthermore, we demonstrate the efficacy of PEI-*f*-CNC for the capture of volatile organic compounds associated with odor. In the first application, we demonstrate the ability of filter cartridges packed PEI-*f*-CNC to reduce ambient volatile fatty acid concentrations at an open-air rendering facility. In the second application, we demonstrate the use of an aqueous suspension of PEI-*f*-CNC in a spray-based method for malodorous VOC reduction. In the broader context of VOC remediation, we focus primarily on the capture of volatile fatty acids and aldehydes that are associated with rendering operations. Rendering plants collect, process, and recycle the by-product stream associated with animal butchering and meat-packing operations. The industry grinds and cooks offal and processes the material into protein meal and refined fats which find use in a variety of applications in agriculture, the oleo-chemical industry, and biodiesel refineries [28]. Nevertheless, the cooking process releases volatile fatty acids and aldehydes resulting from fat degradation that contribute to occasional nuisance odors associated with the industry [29,30,31]. We find that the materials described in this manuscript are capable of capturing gaseous volatile fatty acids and aldehydes from ambient air. 

## 2. Materials and Methods

### 2.1. General 

Solvents and reagents were purchased from MilliporeSigma (Burlington, MA, USA) and VWR International (Radnor, PA, USA), used without further purification. Cellulose microcrystals were purchased from Cellulose Lab (Fredericton, NB, Canada). Commercially available cotton balls were purchased from Walmart supermarket (Bentonville, AR, USA). 

### 2.2. Protocol for Synthesis of CNC from Bulk Cotton 

#### 2.2.1. One-Gram Scale

A 50 mL aqueous solution of 50% H_2_SO_4_ was pre-made and chilled in an ice bath. Then, 1 g of jumbo cotton balls (Walmart, Bentonville, AR, USA) was mechanically processed in a Magic Bullet® blender (Capital Brands, Los Angeles, CA, USA) with a 6 cm × 4 cm cross blade for about 10 min. The cotton was added to a 250 mL round-bottomed flask equipped with a magnetic stir-bar. The flask was cooled in an ice bath and the ice-cold 50% aq. H_2_SO_4_ was added dropwise. The resulting slurry was warmed to 45 °C and stirred for 4 h. The reaction mixture was then filtered through a 40–60 µm pore size fritted funnel, and the filtrate was then centrifuged using an Eppendorf centrifuge 5804 R with 25 mL conical flasks at 8000 rpm for 20 min. The supernatant was decanted, and the wet pellet resuspended in deionized water, and the pH was neutralized by the addition of 6 M aq. NaOH. The neutralized mixture was then centrifuged again, and the wet pellet was dried by freeze drying.

#### 2.2.2. One-Hundred-Gram Scale

**Figure 1:**
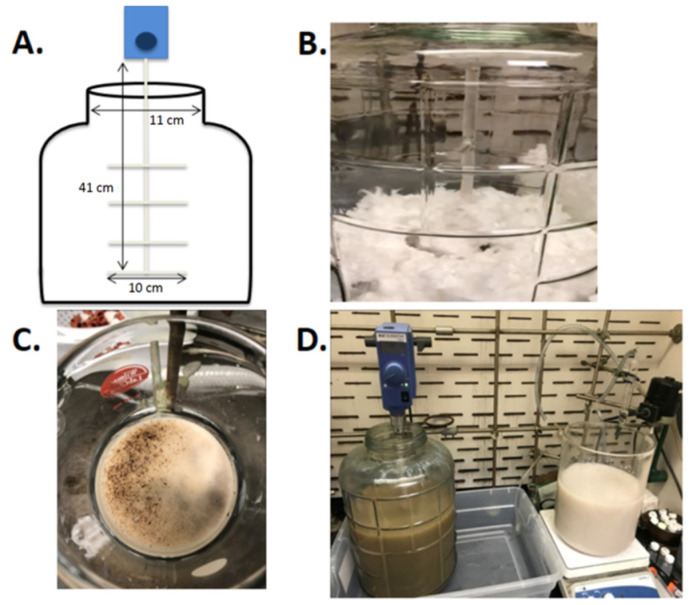
The key steps of the larger scale (i.e., 100 g or 500 g) CNC synthesis: (**A**). apparatus setup, (**B**). mechanically processed cotton, (**C**). removing non-acid hydrolyzed portion of the cotton, (**D**). appearance before (left) and after (right) the work-up.

A 5 L aqueous solution of 50% H_2_SO_4_ was pre-made and chilled in an ice bath. Then, 100 g of jumbo cotton balls (Walmart, Bentonville, AR, USA) were mechanically processed in batches using a Magic Bullet^®^ blender (Capital Brands, Los Angeles, CA, USA) with a 6 cm × 4 cm cross blade for about 10 min. The mechanically processed cotton was then placed in a 25 L wide-mouth glass brewer’s carboy (Fallon Brew Supply, Pendleton, SC, USA). A specially designed glass stir rod was used that appended several perpendicular glass paddles (5 cm) to a vertical glass rod (41 cm) (Figure 1). The mixture was stirred using a mechanical stirrer set at 150–200 rpm. 

The large glass vessel was then cooled in an ice bath in a secondary container. The solution of 50% aq. H_2_SO_4_ was then added drop-wise to the 100 g of mechanically processed cotton while stirring via overhead stirrer. Once the addition of the acidic solution was complete, the ice bath was replaced with a warm water bath. Warm water was continually replenished in order to maintain the water bath at 45 °C in the secondary container. The slurry was left to stir while maintaining the temperature of the water bath at 45 °C for 7 days.

The hydrolysis progress was continuously monitored daily via dynamic light scattering (DLS) measurements (see Section 2.5). DLS monitoring was carried out by first removing a small aliquot from the cellulose slurry, filtering through a 40–60 µm pore size fritted funnel and centrifugation to give a precipitate. Centrifugation for DLS monitoring was performed using an Eppendorf centrifuge 5804 R with 25 mL conical flasks at 8000 rpm for 20 min. The resulting precipitate was then used in the DLS experiment (see Section 2.5). 

Once the desired particle size was reached (ca. 50 nm), the CNC aqueous acidic slurry was centrifuged in 500 mL aliquots for 20 min at 8000 rpm using a SORVALL RC 5C PLUS floor-model centrifuge (ThermoFisher, Waltham, MA, USA) using 500 mL Nalgene bottles with Fiberlite screw top caps. After the centrifugation was completed in batches and the supernatant was decanted, the wet CNC solid was re-suspended in deionized water to achieve a total volume of approximately 4 L. This slurry was filtered through a 40–60 µm pore size fritted funnel. After filtration, the solution was neutralized via the addition of 6 M aq. NaOH (note: approximately 300–400 mL of 6 M aq. NaOH was used to neutralize 4 L of the slurry). The neutralized solution was then centrifuged in 500 mL aliquots, the supernatant was decanted, and the wet CNC pellets were combined and either used directly in the next steps of the synthesis or small samples were freeze-dried to obtain solid CNC samples. Freeze-dried, in-house synthesized CNC was then characterized by DLS, transmission electron microscopy (TEM), powder X-ray diffraction (PXRD), and Attenuated Total Reflection-Fourier Transform Infrared Spectroscopy (ATR-FTIR).

#### 2.2.3. Five-Hundred-Gram Scale

A 500 g batch of mechanically processed cotton was processed similar to the protocol described above in Section 2.2.2 with the following modifications: after mechanical processing of the cotton, 20 L of an ice-cold 50% aq. H_2_SO_4_ solution was slowly added under mechanical stirring, while cooling the 25 L vessel in an ice bath. After the addition of the H_2_SO_4_ solution, the ice bath was replaced with a 45 °C water bath and the mixture was stirred for 7 days. The progress of the reaction was monitored daily by DLS measurements as described above. After 7 days, 5 L of this reaction mixture was removed from the vessel and worked up using the isolation protocol described above in Section 2.2.1 (i.e., centrifugation, resuspension in DI water, filtration, neutralization, and centrifugation). Meanwhile, an additional 5 L of ice-cold 50% aq. H_2_SO_4_ was slowly added to the reaction vessel and the slurry was allowed to stir for an additional 7 days at 45 °C (with daily monitoring by DLS measurements). After this time, the remaining reaction mixture was split in half and worked up using the isolation protocol described above in Section 2.2.2.

### 2.3. TEMPO-Mediated Oxidation of CNCs (100 g Scale)

**Scheme 1:**
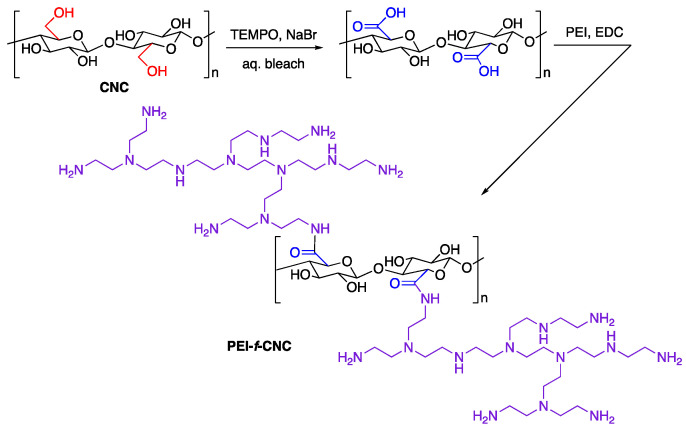
Two-step modification of CNC to CNC-*f*-PEI by TEMPO oxidation/carbodiimide coupling sequence.

Scheme 1 depicts the synthetic sequence for converting CNCs to PEI-*f*-CNCs. A 1 kg sample of an aqueous CNC slurry (i.e., 10–12 wt.% CNC in H_2_O) was suspended in 5 L of water in a 25 L wide-mouth brewer’s carboy (Fallon Brew Supply, Pendleton, SC, USA). TEMPO (10 g, 63 mmol), sodium bromide (1.25 g, 12 mmol), and 12% aq. NaClO solution (240 g, 387 mmol) was added to the resulting solution in order to initiate the TEMPO-mediated oxidation. This reaction mixture was stirred at room temperature for 10 days. A couple of hours after the addition of the reagents for the oxidation step, the pH of the solution was checked and maintained at pH 10 by the addition of a 0.5 M NaOH solution (approximately 5–10 mL every 24 h) throughout the course of the reaction. After 10 days, the pH of the resultant suspension was adjusted to 2.5 by adding approximately 50–75 mL 0.1 M aq. HCl in order to neutralize the sodium carboxylate functional groups formed during the oxidation. The neutralized suspension was left at rest for 1–2 days while monitoring and maintaining the pH at 2.5. The resulting mixture was used for the next step of the synthesis (i.e., PEI functionalization) without further purification.

### 2.4. PEI-Functionalization (100 g Scale)

1-Ethyl-3-(3-dimethylaminopropyl)carbodiimide (EDC, 110 g, 574 mmol) and 375 mL of a 50% wt. % aq. polyethylenimine solution (PEI, M_w_ ca. 1300) were added to the mixture generated from the TEMPO oxidation step and the resulting mixture was stirred at room temperature for 10 days. The resultant slurry was purified using dialysis membrane purification (Spectra/Por^®^2 dialysis membrane standard RC tubing; MW cut-off: 12–14 kD; flat width: 45 mm; diameter: 29 mm)). The water bath for the dialysis purification was exchanged with fresh deionized water every 24 h until no significant pH change in the water was observed. (Note: the above protocols in Section 2.3 and Section 2.4 were also successful for the functionalization of CNC with PEI on 1 g and 10 g scales by simply adjusting the proportion of the solvent and reagents).

### 2.5. Characterization of PEI-f-CNC Materials

#### 2.5.1. Dynamic Light Scattering (DLS) Measurements

A Brookhaven NanoBrook Omni instrument (Holtsville, NY, USA) was used to perform the DLS measurements. A small aliquot of CNC slurry was first filtered through a 40–60 µm pore size fritted funnel. The filtrate was then centrifuged for 20 min, then diluted with de-ionized water to obtain a uniform, transparent solution prior to DLS analysis. The parameters used in the DLS measurements were: set duration: 180 s; equilibration time: 180 s; total measurements: 4; time interval between measurements: 10 s; dust cut off: 50.00; cell type: Bi-SCP; angle: 90 degree scattering for smaller particles and forward scattering for larger particles; refractive index of particles: 1.59; particle shape: uniform spheres; baseline normalization: auto; size distribution: used CONTIN for broad unimodal distribution. 

#### 2.5.2. Transmission Electron Microscopy (TEM)

H7600 and HT7830 microscopes (Hitachi High-Tech, Tokyo, Japan) operated at 100 kV and 120.0 kV voltage were used to collect transmission electron microscopy (TEM) images. A moderately turbid CNC slurry solution was prepared for TEM analysis by dissolving about 50 mg of freeze-dried CNC in 5–10 mL of deionized water in a 20 mL glass vial. A TEM grid was placed on a square piece of parafilm and one drop of CNC solution was placed on top of the dark side of the TEM grid using a 1 mL plastic dropper. The TEM grid was positioned on a square piece of parafilm followed by the addition of one drop of 3% uranyl acetate. Uranyl acetate staining was carried out over 30 s at room temperature. This TEM grid was dried in a desiccator with the TEM grid holder for 24 h prior to TEM analysis. For more rapid analysis, the TEM grid can be placed on a piece of aluminum foil followed by gentle heating (ca. 40 °C) on a hot plate up to 1 h and then placed in a desiccator with the TEM grid holder prior to TEM analysis.

#### 2.5.3. Powder X-ray Diffraction of Commercial and in-House CNC

Powder X-ray diffraction was carried out using a Rigaku Ultima IV diffractometer (Tokyo, Japan) with CuKα radiation (λ = 1.5406 Å) at 0.02° intervals at a rate of between 0.2–1°/min from a 2θ range of 5° to 65°. PXRD-PDXL software was used to analyze the composition and the purity of the product. Origin 8 software (OriginLab, Northampton, MA, USA) was used to plot the powder patterns. The three main diffraction peaks corresponding to cellulose type I were used to analyze the crystallinity percentage. These peaks can be identified as a sharp high peak at around 2θ = 22.5° and weaker peaks closer to 2θ = 14.8°, 16.3° which are assigned to the 200, 110, and $1\bar{1}0$ crystallographic planes of the monoclinic cellulose Iβ lattice, respectively [32]. The diffraction pattern was deconvoluted into crystalline and amorphous peaks. The integrated intensity provided the area under each of those peaks from which the percent crystallinity was calculated using Equation (1), where I is the integrated intensity:

(1) $$\%\;\mathrm{crystallinity}=\left(I_{\mathrm{cryst}}\right)/\left(I_{\mathrm{cryst}}{+I}_{\mathrm{amorph}}\right)$$

#### 2.5.4. Attenuated Total Reflection-Fourier Transform Infrared Spectroscopy (ATR-FTIR)

Infrared spectroscopy data were collected using an ATR-FTIR instrument (Shimadzu, IRAffinity-1S instrument with MIRacle 10 single reflection ATR accessory, Tokyo, Japan) and scanned over the range of 400 to 4000 cm^−1^.

#### 2.5.5. Thermo Gravimetric Analysis (TGA)

TGA data were collected using TGA instrument SDT Q600 V20.9 Build 20 (Hitachi, Tokyo, Japan) over the temperature range of 200–800 °C, with a ramp rate of 10 °C/min and under a nitrogen flow rate of 100 mL/min.

### 2.6. CNC-f-PEI Cartridge Preparation 

**Figure 2:**
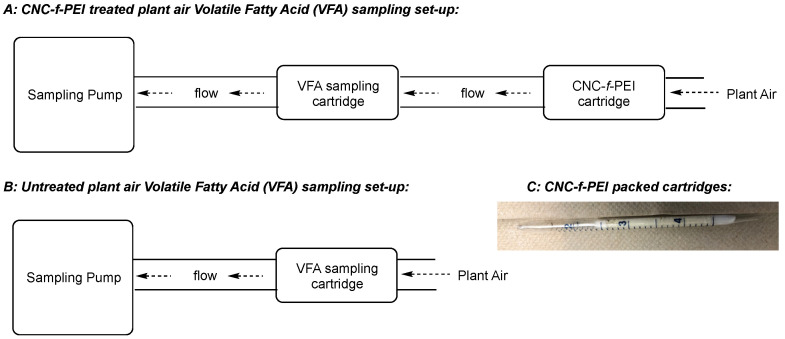
In-plant sampling setup for evaluation of CNC-*f*-PEI packed cartridges for VOC capture. (**A**). Sampling setup for VFA sampling of CNC-*f*-PEI treated air. (**B**). Sampling setup for VFA sampling of untreated plant air. (**C**). Photograph of CNC-*f*-PEI packed cartridges.

Sample cartridges were prepared by loading ca. 0.5 g of CNC-*f*-PEI between two loosely packed glass wool plugs in a glass tube (7 mm inner diameter), consistently achieving a CNC-*f*-PEI bed length of ca. 4 cm. The cartridges were then sealed by heating the tips of the glass over a Bunsen burner until the glass became malleable enough to seal off each end of the cartridge with gentle twisting (see Figure 2C). 

### 2.7. In-Plant Sampling Experiment:

The CNC-*f*-PEI cartridges were assessed for volatile fatty acid (VFA) abatement in an open-air rendering facility in the Central Valley region of California, USA [29] using the experimental design depicted below in Figure 2 and the accompanying procedure. The sampling equipment was positioned in the plant near the raw material pile and the cookers. Plant air treated with CNC-*f*-PEI cartridges for VFA abatement was sampled in triplicate using an SKC universal air sampling pump (SKC Ltd., Dorset, UK) that was plumbed in sequence to a commercially available KOH-impregnated silica gel VFA sampling cartridge followed by a PEI-*f*-CNC packed cartridge using short lengths of tubing (Figure 2A). Untreated plant air was sampled by plumbing just the KOH-impregnated silica gel cartridge with a short length of tubing (Figure 2B). The VFA samples were collected at a 700 mL min^−1^ flow rate for 100 min.

After the sampling period, the VFA sampling cartridges were sealed with plastic caps and stored in zip-top bags at 0 °C during transport to a commercial atmospheric sampling laboratory (Atmospheric Analysis & Consulting, Inc, Ventura, CA, USA), where they were processed using GC/MS analysis following the laboratory’s standard operation procedures. Data reports were received that reported in relation to the method reporting limit (MRL) for the assay. The parts-per-billion (ppb) level concentrations of each of the detected VFAs were averaged and standard deviation was calculated.

### 2.8. General Procedure for the Spray Experiment of with Aqueous Suspension of PEI-CNC Materials

**Figure 3:**
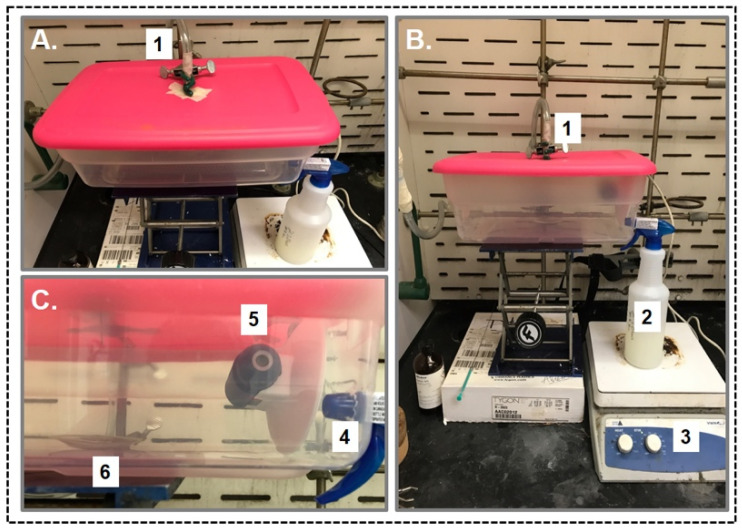
Spray experiment setup. (**A**): top view, (**B**): side view, (**C**): aeroqual probe view, 1: nitrogen line to produce hexanal vapors, 2: PEI-*f*-CNC aqueous solution with a stir bar, 3: stir plate, 4: delivering of the PEI-*f*-CNC solution, 5: aeroqual probe, 6: hexanal liquid on the watch glass.

A spray chamber was constructed as outlined in Figure 3. An Aeroqual VOC probe (Auckland, New Zealand) was attached inside the spray chamber (HDPE rectangular container with sealable lid, dimensions: 17.8 cm H × 39.4 cm W × 55.9 cm L; 20 L volume), then the chamber was closed and allowed to equilibrate until the Aeroqual probe read a ppm value in between 0 and 0.50 ppm for VOCs. This value was taken as the baseline value in each spray experiment attempt. Once the VOC reading fell to within the above range, 0.6–0.8 mL of hexanal was introduced using a syringe from the top of the chamber onto a watch glass placed in the middle of the chamber. The chamber was then provided a constant dry nitrogen flow (~200 cycles/min as measured using Bel-Art flow indicator (Wayne, NJ, USA) from the top of the watch glass. After the VOC ppm value reached 7–9 ppm under nitrogen flow and stabilized, the aqueous suspension of the CNC-*f*-PEI was sprayed into the chamber (note: 3 sprays corresponded to 2 mL of CNC-*f*-PEI suspension). Then, the VOC ppm reading was recorded until the ppm value stabilized (typically after about 8–10 min). All of the spray experiments were performed in triplicate. The spray solution was continuous stirred during the entire time of the experiment to prevent settling of the CNC-*f*-PEI suspension. Control spray experiments were conducted using deionized water (i.e., without added CNC-*f*-PEI). Untreated hexanal VOC control samples were measured to acquire the remediation % by water and Aeroqual probe error %, respectively. 

## 3. Results and Discussion

### 3.1. Scaled Synthesis of CNC from Bulk Cotton

A requirement of a successful in-house CNC synthesis from bulk cotton was to maintain the particle size of the resulting CNC below or near 100 nm in diameter. In order to develop an optimized protocol for the CNC synthesis in our laboratory, we conducted a large number of 1 g scale acid hydrolysis experiments with different types of cotton (Table 1). During this initial grounding study, we explored aqueous hydrochloric acid and aqueous sulfuric acid as the hydrolyzing agents. The particle size was measured using dynamic light scattering (DLS) in order to rapidly optimize the protocol. The cellulose microcrystals (CMC), store-bought cotton balls (Cotton-A) and mechanically processed cotton (Cotton-B, which was processed in a Magic Bullet^®^ mini blender for about 10 min) were hydrolyzed with 2.5 M aq. HCl for 0.5 h to 4 h and provided 93 nm, 560 nm, and 1800 nm particles, respectively (entry 1–3). The mechanically processed cotton balls (Cotton-B) were treated with 50% aq. H_2_SO_4_ at room temperature and 45 °C to provide 175 nm and 52 nm particles, respectively (entry 4 and 5).

Nevertheless, we noticed that upon freeze drying of the slurry of the 52 nm particles, the resulting solid materials exhibited a much larger particle size (375 nm) by DLS measurement after resuspension in fresh deionized water, which suggested that freeze drying of this sample caused significant agglomeration of the nanocrystals into larger particles. Lowering the concentration of H_2_SO_4_ to a 25% aqueous solution yielded much larger particles (i.e., 9970 nm, entry 6). Attempting to increase the temperature of the acid hydrolysis while maintaining the H_2_SO_4_ concentration at 50% in water resulted in slightly higher particle size (77 nm, entry 7). The CMC, Cotton-A, and Cotton-B samples were then treated with a slightly higher concentration of H_2_SO_4_ (i.e., 64% in water) at 45 °C with the hope of shortening the hydrolysis time. However, the resultant cellulose slurries showed polydisperse particle sizes over the range of 90–430 nm (entry 8–10). Since the 50% aq. H_2_SO_4_ at 45 °C for 4 h afforded the smallest particle size of CNC (entry 5, highlighted in bold), it was selected as the optimized hydrolysis condition for larger scale CNC synthesis.

During the optimization process of CNC synthesis, we noticed that the pH of the slurry plays a significant role in promoting the agglomeration of the CNC particles upon freeze drying. If the CNC slurry was freeze-dried after the acid hydrolysis, the particles tended to agglomerate and form much larger particles (i.e., >1000 nm at pH = 2.7), whereas if the CNC slurry was neutralized by the addition of aqueous 6 M sodium hydroxide after the acid hydrolysis, the particle size remained below 100 nm (i.e., 60 nm at pH = 6.9), even after the freeze-drying process. 

We then turned towards the larger scale CNC synthesis using the conditions outlined in entry 5. Ultimately, we found that stirring a slurry of 100 g of mechanically processed cotton balls in a 50% aqueous H_2_SO_4_ solution over the course of 7 days at 45 °C resulted in a slurry of CNC material with an average diameter of approximately 50–100 nm as judged by DLS measurements.

Using a similar approach, a 500 g scale reaction was successfully performed. The final results of these different experimental scales are illustrated in Table 2. The experiment of the 100 g scale provided to give CNC particles with a hydrodynamic size of 60 nm in 50% yield and the 500 g scale reaction provided CNCs with an average hydrodynamic size of 177 nm in 65% yield (entry 2 and 3). This shows the applicability of the protocol in the larger scale synthesis of CNCs.

**Figure 4:**
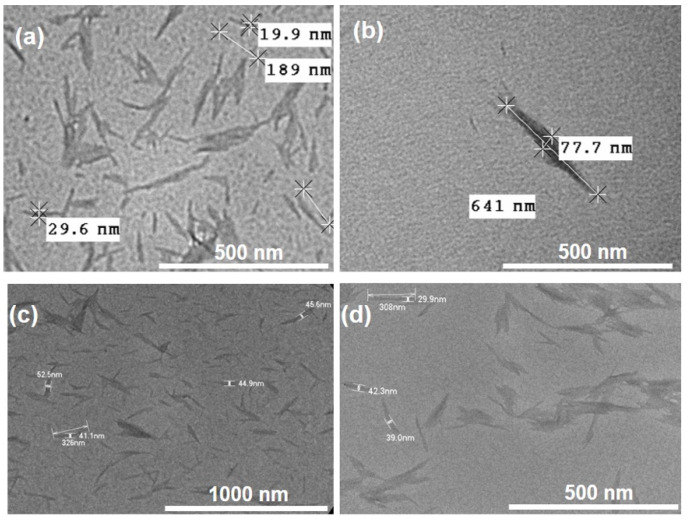
TEM images of CNCs at different magnifications which represents the crystalline, needle-like structures; (**a**) commercial CNC, (**b**) in-house CNC from 1 g scale synthesis, (**c**,**d**) in-house CNC from 100 g scale synthesis.

The slurries resulting from the CNC synthesis were further characterized by transmission electron microscopy (TEM) (Figure 4). The width of the cellulose nanorods captured in TEM images were in good agreement with the hydrodynamic size judged by DLS. Furthermore, it suggests that both the in-house CNC that we synthesized and the commercial CNC (30 nm) have the same needle- or rod-like crystalline structures and they are both in comparable size range. In our hands, DLS measurements on aqueous suspensions of commercial and in-house CNC samples often correlated more closely to the observed diameter (i.e., width) of the CNC needles than their length as judged by TEM.

**Figure 5:**
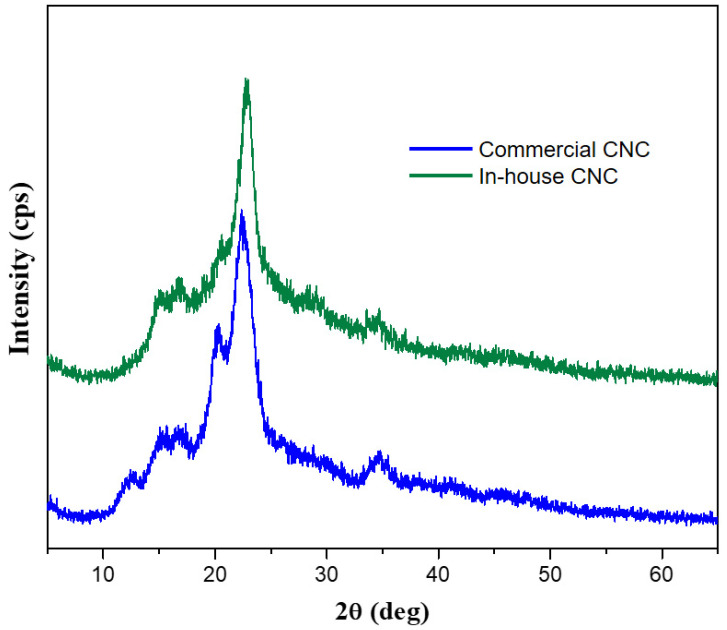
PXRD patterns of commercial and in-house CNC.

We also evaluated the crystallinity of the resulting cellulose nanocrystals by means of powder X-ray diffraction experiments (Figure 5). Commercial and in-house CNC samples both showed three main diffraction peaks corresponding to cellulose type I [32], which are a sharp high peak at around 2θ = 22.5° and weaker peaks closer to 2θ = 14.8°, 16.3°. These diffraction peaks can be assigned to the 200, 110, and $1\bar{1}0$ crystallographic planes of the monoclinic cellulose Iβ lattice, respectively. Due to the more prominent crystalline peaks at 12.4 and 20.3 degrees in the commercial CNC, the crystallinity of the commercial CNC was found to be slightly higher (i.e., 45.5%) than the in-house synthesized CNC (i.e., 37.1%).

Thus, we developed a reliable and inexpensive laboratory protocol to synthesize cellulose nanocrystals (CNC) from bulk cotton. Moreover, this protocol was further developed to perform an even larger-scale synthesis of CNC (i.e., up to 500 g) while achieving a reasonable size range (100–200 nm) of CNC particles in good yield.

### 3.2. Scaled Synthesis of PEI-f-CNC from In-House Prepared CNC

Our next objective in this study was to evaluate our previously developed protocol [22] in the context of a larger-scale synthesis of PEI-*f*-CNC from unmodified CNCs. Thus, we investigated the use of a 100 g slurry of our in-house synthesized CNC to perform the PEI functionalization protocol at a 100-fold larger scale than previously attempted. PEI modification of CNC results from a two-step modification sequence that involves TEMPO-catalyzed oxidation of the C-6 carbinol of CNCs followed by carbodiimide coupling of poly(ethylenimine) mediated by the water soluble carbodiimide, EDC (Scheme 1, vide supra). We found that both of these transformations could be carried out on a 100 g scale to provide ready access to PEI-*f*-CNC (see Materials and Methods section for details.) 

**Figure 6:**
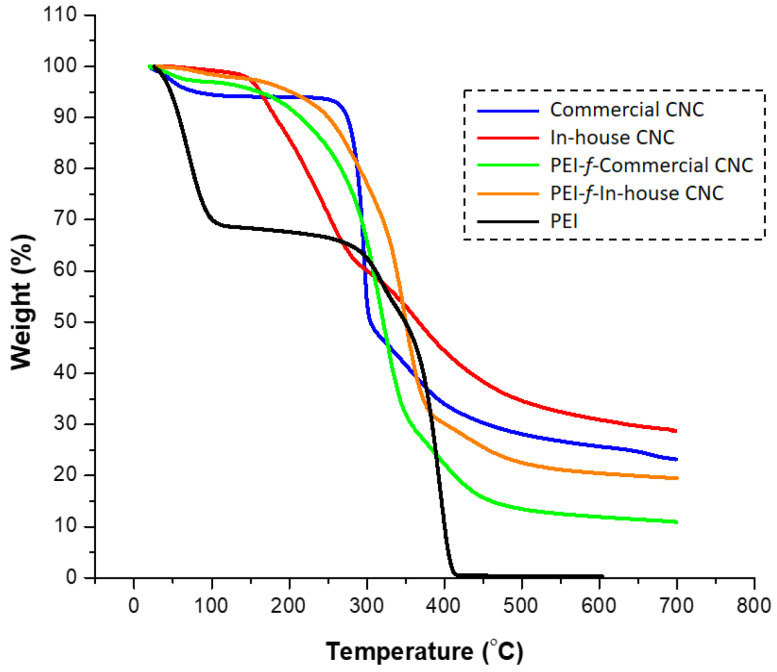
TGA curves of the non-functionalized and PEI-functionalized materials of commercial CNC and in-house synthesized CNC.

A comparison of the TGA data arising from the PEI-*f*-CNC materials resulting from commercially available CNC and in-house synthesized CNC and related controls is shown in Figure 6. The commercial CNC showed a rapid thermal weight loss around at 265 °C, whereas the in-house synthesized CNC showed a gradual thermal weight loss from 150 °C. The natural source of the cellulose used to generate the commercial CNC samples that were analyzed in this study was not disclosed by the manufacturer, but it is likely that that it did not originate from cotton. Rather, wood pulp is the most common commercial source of cellulose nanocrystals [18]. We have noted previously that the primary natural source of cellulose micro and nanocrystals can dramatically affect the structural parameters of the resulting isolates in terms of overall morphology, surface area, porosity, and crystallinity [26]. We hypothesize that these structural differences in cellulose isolates originating from different primary sources can manifest themselves in observable differences in degradation profile samples when analyzed by TGA and slight differences in the FT-IR spectra of specific isolates. It is also possible that the specific conditions of the degradation process used to prepare the CNCs may result in different surface chemistry on the particles, but this is harder to judge. However, the steady decrease of the weight percentage over temperature seen in PEI-*f*-commercial-CNC and PEI-*f*-in-house-CNC clearly suggest that the materials are successfully functionalized. 

**Figure 7:**
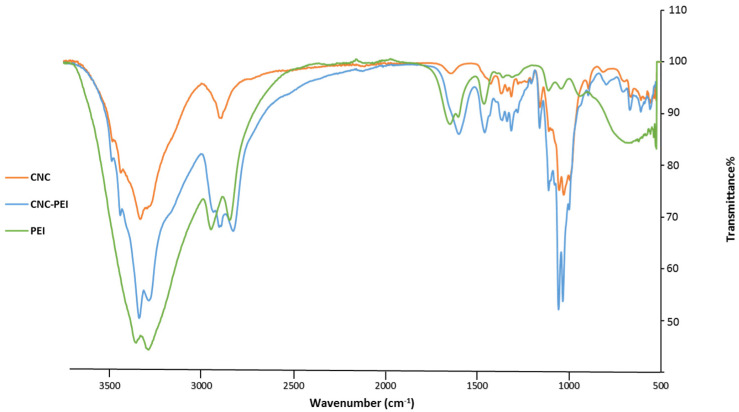
ATR-FTIR spectra of the non-functionalized and PEI-functionalized materials of commercial CNC and in-house synthesized CNC. Orange line: FT-IR spectrum of in-house prepared CNCs; blue line: FT-IR of PEI-*f*-CNC prepared from in-house generated CNC; green line: FT-IR spectrum of PEI.

Next, we evaluated the FT-IR spectra (Figure 7) of the in-house prepared CNCs (orange line), and the PEI-*f*-CNC material (blue line). The FT-IR spectrum of commercial PEI was also collected (green line). Diagnostic peaks for the unmodified cellulose nanocrystals include C–C, C–OH, C–H ring, and side group vibrations, as well as C–O–C glycosidic ether bands over the range of 1000–1100 cm^−1^, along with CH stretching bands (ca. 2900 cm^−1^) and OH stretching bands (ca. 3300 cm^−1^) [26]. These signals are apparent in the spectrum PEI-*f*-in-house CNC along with the emergence of strong NH-bending peaks observed over the range of 1500 to 1750 cm^−1^ in the ATR-FTIR spectrum of confirmed the successful PEI grafting (Figure 7, blue line) [22].

**Figure 8:**
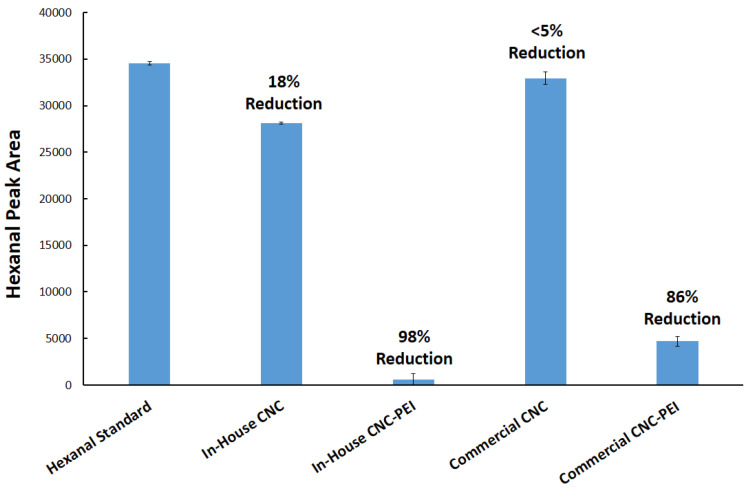
Hexanal vapor capture studies of the non-functionalized and PEI-*f*-CNC materials arising from commercial CNC and in-house synthesized CNC.

After the confirmation of successful PEI functionalization from the in-house synthesized CNC, we then turned towards assessing its VOC vapor capture capabilities using our standard GC assay [19,22]. Figure 8 depicts the hexanal vapor capture studies with non-functionalized CNC and PEI-*f*-CNC materials resulting from commercial CNC and in-house synthesized CNC starting materials. Based on the data presented in Figure 8, it is clear that the in-house-prepared PEI-*f*-CNC material is capable of capturing a target VOC, hexanal with 98% reduction (cf. 86% reduction with PEI-*f*-CNC resulting from commercial CNC material). These VOC studies were conducted according to our previously published protocol by adding a small amount (i.e., 10 µL) of hexanal into a 1 mL gas chromatography vial, subsequently placing the nanomaterial (50 mg) on a tissue paper towel (i.e., Chemwipe) in the headspace of the vial and capping with a rubber septa. The headspace of the vial was then analyzed via GC peak area studies [19,22]. The significantly enhanced capability of PEI-*f*-CNC to capture aldehyde VOCs as compared to unmodified CNC materials is due to the availability of significant amounts of amine functional groups on the surface of the modified material, which we have shown are capable of forming covalent interactions with aldehyde VOCs by means of imine formation [19].

**Figure 9:**
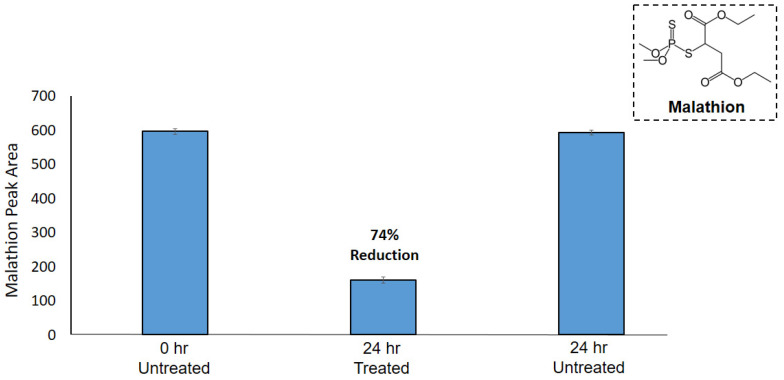
Pesticide remediation studies with CNC-*f*-PEI: degradation of Malathion in dichloromethane.

Furthermore, the pesticide remediation capabilities of the fully in-house prepared PEI-*f*-CNC were also evaluated. These studies were performed with the well-known pesticide malathion using GC peak area analysis using our previously published method [27]. Malathion remediation experiments were set up by adding the PEI-*f*-CNC materials directly into a solution of malathion in dichloromethane followed by filtration and analysis of the amount of malathion present in the solution using GC after 24 h. The results of these studies shows that the malathion solutions treated with the fully in-house prepared PEI-*f*-CNC materials can reduce the concentration of pesticide in solution by 74% after 24 h, suggesting that the materials are efficient in pesticide remediation as well (Figure 9). We have shown previously that the degradation of malathion by PEI-*f*-CNC is due to a clean β-elimination facilitated by the amines on the surface of the material, thus accounting for the superior performance of PEI-*f*-CNC as compared to unmodified CNC [27]. 

After successfully achieving the large-scale synthesis of CNCs from bulk cotton (up to 0.5 kg scale) and subsequent modification to PEI-*f*-CNC (up to 100 g scale), we next leveraged our access to larger amounts of material to evaluate the utility of PEI-*f*-CNC material for odor and VOC abatement in two applications: use as a packed bed filter agent and use as a spray-based odor-remediation strategy. 

### 3.3. Application of PEI-f-CNC Packed Bed Filter Cartridges for VOC Remediation

First, we set out to evaluate the performance of packed cartridges (Figure 2C, vide supra) of PEI-*f*-CNC for the remediation of volatile fatty acids (VFA) from ambient air surrounding an open-air rendering plant [29]. Using sampling pumps, the concentration of untreated VFAs was measured over a 100 min sampling period in the plant (Figure 2A). Simultaneous measurements of plant air treated by passing through a 4 cm packed-bed cartridge of PEI-*f*-CNC were also collected (Figure 2B). PEI-*f*-CNC treated air samples were collected in triplicate. The results were averaged, and the standard deviation was calculated. The resulting data are highlighted in Table 3. 

Eleven distinct fatty acids were detected in the ambient, untreated plant air in concentrations ranging from 0.51 to 290 ppb. Generally, the more volatile fatty acids were detected in higher concentrations. The three constituents with the highest concentrations were acetic acid (290 ppb), butyric acid (250 ppb), and propanoic acid (190 ppb), and are all commonly encountered malodorous VOCs associated with fat degradation processes common to rendering operations [29,30,31,33]. Remarkably, in parallel experiments where ambient plant air was filtered through packed-bed cartridges containing PEI-*f*-CNC prior to entering the sampling cartridge, the detectable levels of VFAs were dramatically lower. Indeed, nine of the eleven VFAs present in untreated plant air were undetectable after passing through PEI-f-CNC cartridges, including acetic acid, which was the most prevalent constituent in untreated plant air. Only propanoic acid and butyric acid were present in detectable levels after passing through PEI-*f*-CNC cartridges. Propanoic acid concentrations were reduced to 2.03 ± 0.55 ppb (99% reduction) and butyric acid was reduced to 2.53 ± 0.11 ppb (99% reduction). Concentrations of all other VFAs were below the limit of detection for the analytical method. These data clearly indicate the excellent performance of PEI-f-CNC cartridges for the removal of malodorous VFA constituents form rendering plant air. We hypothesize that the capture of VFAs with PEI-*f*-CNC is facilitated by the rapid formation of ammonium carboxylate salts upon reaction of the VFA with surface-bound amine groups on the nanoparticles [19,23]. 

### 3.4. Application of PEI-f-CNC in A Spray-Based Delivery Method for VOC Remediation

Finally, we sought to evaluate whether aqueous slurries of PEI-*f*-CNC could be used as a spray-based strategy for removing malodorous VOCs from ambient air. In order to carry this out, we constructed a laboratory-scale spray chamber equipped with a commercially available remote VOC sensor (Figure 3, vide supra). For the spray experiments, we prepared slurries of PEI-*f*-CNC in deionized water at 25 g/L and 12.5 g/L concentrations.

In these experiments, a nitrogen line was used to produce hexanal vapors after hexanal liquid was placed on the watch glass in the center of the chamber. After the VOC level of the chamber reached equilibrium, an aqueous suspension of the PEI-*f*-CNC materials was then delivered into the sealed chamber from the side while reading the ppm value corresponding to the hexanal vapors using an Aeroqual VOC probe (Auckland, New Zealand). 

**Figure 10:**
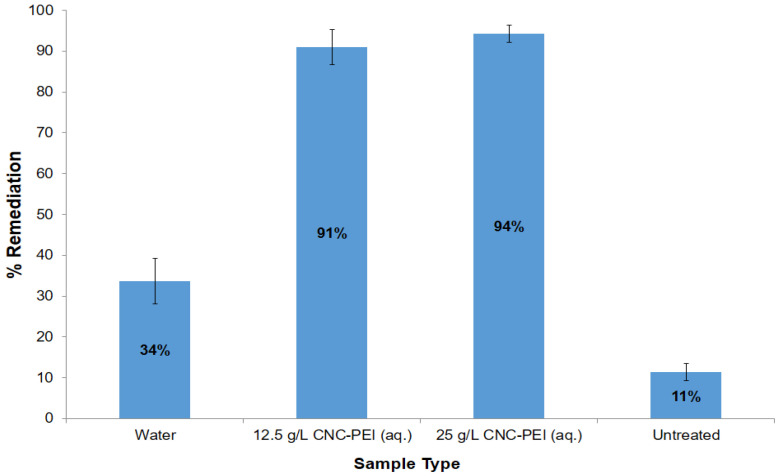
Hexanal remediation from the spray experiment.

The results from this spray study showed that an aqueous suspension of the PEI-f-CNC materials (12.5 g/L and 25 g/L slurry) can reduce hexanal vapors by ≥ 90% (Figure 10). In comparison, spraying deionized water without CNC-*f*-PEI resulted in a significantly lower 34% reduction of the hexanal vapors. As a control, we noted that the VOC level of the untreated chamber fluctuated by just 11% over the same time period. 

**Figure 11:**
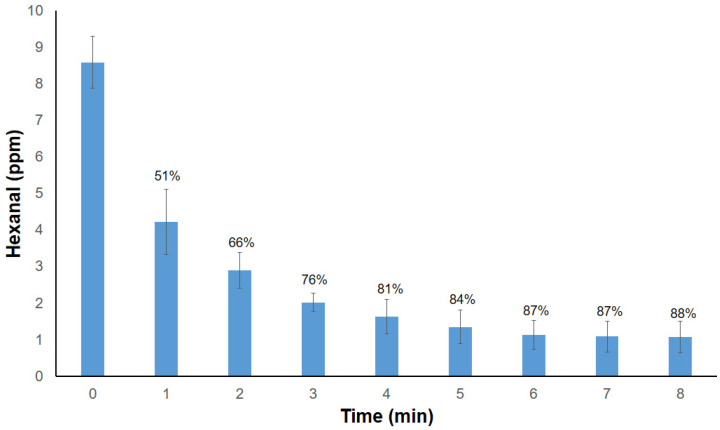
Hexanal remediation over time from the spray experiment.

Next, we carried out a time course experiment, wherein we monitored the change in concentration of the target VOC, hexanal, over time using a 25 g/L suspension of PEI-*f*-CNC. This study demonstrated that hexanal vapors are reduced by 50% within the first minute of treatment and then it slows down gradually over a period of 8–10 min (Figure 11).

**Figure 12:**
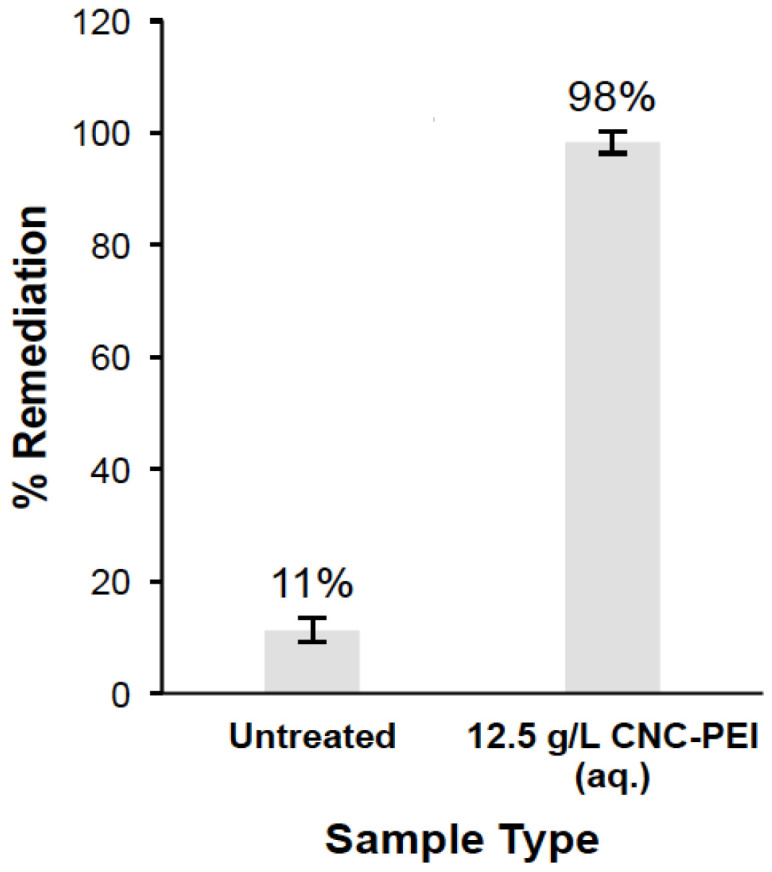
Remediation of 1:1 mixture of hexanal/hexanoic acid with 12.5 g/L aqueous suspension of PEI-CNC.

In a final experiment, we sought to evaluate whether the PEI-*f*-CNC suspension could remediate odors associated with both an aldehyde and fatty acid (carboxylic acid) constituent simultaneously. In this experiment, a 12.5 g/L PEI-*f*-CNC aqueous suspension was used to treat a 1:1 mixture of hexanal and hexanoic acid. In the event, the PEI-*f*-CNC slurry effectively removed the two compounds from the atmosphere of the chamber in 98% efficiency (Figure 12).

## 4. Summary and Conclusions

We were able to develop a reliable, scalable synthesis of cellulose nanocrystals on 1 g and 100 g scale in our laboratory (i.e., hydrodynamic size of 52 and 60 nm, respectively). Furthermore, processing 500 g of cotton resulted in the isolation of a mixture of micro and nanocrystalline cellulose (i.e., average hydrodynamic size of 177 nm). We successfully prepared PEI-*f*-CNC at 1 g, 10 g, and 100 g scales. We further confirmed that the PEI-*f*-CNC materials resulting from in-house-made CNC from bulk cotton exhibit the same remediation efficiency as smaller scale batches of PEI-*f*-CNC materials prepared from commercial CNC sources. Additionally, we demonstrated that the PEI-*f*-CNC materials are capable of significantly reducing malodorous volatile fatty acid concentrations from ambient plant air at an open-air rendering plant. Finally, we conducted preliminary, laboratory-scale experiments that demonstrate the ability of aqueous slurries of PEI-*f*-CNC to reduce VOC levels by means of a spray-based delivery. The successful preparation of CNCs and PEI-*f*-CNCs at larger scales sets the stage for pilot-scale syntheses of these materials to access kilogram quantities. The successful small-scale validation of the materials as packed bed filters or aqueous slurries will also be explored at a larger scale in the future.

## Figures and Tables

**Table 1:** Optimization of the cellulose nanocrystal synthesis.

Entry	Cellulose Source	Acid	Temperature (°C)	Time (h)	Diameter (nm) ^4^
1	CMC ^1^ (20 um)	2.5 M HCl	100	0.5	93
2	Cotton-A ^2^	2.5 M HCl	100	0.5	560
3	Cotton-B ^3^	2.5 M HCl	100	4	1800 ^5^
4	Cotton-B	50% H_2_SO_4_	20	4	175
5	Cotton-B	50% H_2_SO_4_	45	4	52 (375) ^6^
6	Cotton-B	25% H_2_SO_4_	45	4	9970
7	Cotton-B	50% H_2_SO_4_	60	4	77
8	CMC (20 µm)	64% H_2_SO_4_	45	1	432
9	Cotton-A	64% H_2_SO_4_	45	1	91
10	Cotton-B	64% H_2_SO_4_	45	1	416

^1^ CMC (20 µm) = cellulose microcrystals filtered through 20 µm pore size filter. ^2^ Cotton-A = unprocessed store-bought cotton balls. ^3^ Cotton B = mechanically processed store-bought cotton balls. ^4^ As judged by DLS measurements; 90° scattering detection used for small particles. ^5^ As judged by DLS measurements; forward scattering detection used for larger particles. ^6^ after freeze drying.

**Table 2:** Results of the different experimental scales of CNC synthesis.

Entry	Scale	Hydrodynamic Size (nm) ^1^	Yield (%)
1	1 g	52	20
2	100 g	60	50
3	500 g	177	65

^1^ as judged by averaged DLS measurements.

**Table 3:** Capture of VFAs emitted from a rendering plant using packed-bed cartridges of PEI-f-CNC.

VFA	Untreated (ppb)	Treated (ppb)	% Reduction
acetic acid	290	ND ^1^	>97%
propanoic acid	190	2.03 ± 0.55	99%
isobutyric acid	31	ND	>98%
butyric acid	250	2.53 ± 0.11	99%
2-methylbutanoic acid	20	ND	>97%
isovaleric acid	15	ND	>96%
valeric acid	18	ND	>97%
isocaproic acid	19	ND	>97%
caproic acid	7.2	ND	>93%
enanthoic acid	0.51	ND	>8%
caprylic acid	0.55	ND	>24%

^1^ ND = not detected.

## Data Availability

All pertinent data are included in the body of the manuscript.
